# Spontaneous bilateral tubal ectopic pregnancy: a case report

**DOI:** 10.11604/pamj.2021.38.395.28771

**Published:** 2021-04-23

**Authors:** Michael Nyakura, Felix Godwin Mhlanga, Mugove Madziyire, Sitshengiso Matshalaga

**Affiliations:** 1 Department of Obstetrics and Gynaecology, Victoria Falls Hospital, Victoria Falls, Zimbabwe,; 2 Academic Department of Obstetrics and Gynaecology, College of Health Sciences, University of Zimbabwe, Harare, Zimbabwe,; 3 Department of Histopathology, College of Health Sciences, University of Zimbabwe, Harare, Zimbabwe

**Keywords:** Spontaneous, bilateral, ruptured, ectopic pregnancy, case report

## Abstract

Bilateral tubal ectopic pregnancy is a very rare form of extra-uterine pregnancy with high maternal morbidity and mortality if intervention is delayed. We report the case of a 27-year-old para 2 gravida 3 patient who presented in haemorrhagic shock after delayed diagnosis of ectopic pregnancy. An ultrasound scan noted a right tubal ectopic pregnancy. At laparotomy, bilateral ruptured tubal ectopic pregnancy was encountered and bilateral salpingectomy was done as both tubes were not salvageable. She recovered completely postoperatively and histology confirmed bilateral tubal ectopic pregnancies. Bilateral tubal ectopic pregnancy may not be easily diagnosed on a scan; hence vigilance at surgery is critical to prevent maternal mortality.

## Introduction

The incidence of ectopic pregnancy worldwide is 1-2% [1]. Similarly, in a study done at Harare and Parirenyatwa Hospitals, Zimbabwe, the incidence was 1.12% [2]. Ruptured ectopic pregnancy is an important cause of maternal morbidity and mortality. Approximately 97% of extra-uterine pregnancies occur in the fallopian tubes [3]. Bilateral tubal ectopic pregnancy (BTP) is a very rare type of ectopic pregnancy. The early reported frequency of bilateral ectopic pregnancy has been estimated at 1/200,000 uterine pregnancies and 1/725-1/1580 ectopic pregnancies [4]. The occurrence has tripled in the last decades with most cases being associated with assisted reproduction techniques (ART), use of intrauterine contraceptive devices (IUD), pelvic inflammatory disease (PID) and history of previous ectopic pregnancy or tubal surgery [5].

The criteria for diagnosis of BTP were first suggested by Fishback who declared that there should be a description of the fetuses or fetal parts as well as of placental material in both tubes [4]. This was later revised by Norris who stated that microscopic demonstration of chorionic villi in each tube was sufficient for the diagnosis [4]. We report a case of spontaneous BTP that we managed at Parirenyatwa Group of Hospitals, Harare, Zimbabwe in January 2017.

## Patient and observation

Mrs FM, 27-year-old, para 2 gravida 3, a widow and massage therapist, presented with a two week history of lower abdominal pain and per vaginal bleeding after 14 weeks of amenorrhea. The bleeding was minimal but pain was progressively severe associated with symptoms of anaemia. She had consulted a general practitioner two weeks earlier when her symptoms started. A pregnancy test was positive and an ultrasound scan was requested which she could not afford and returned home hoping for spontaneous resolution. She then presented to our casualty department with worsening of symptoms, general body weakness and difficulty mobilising. This time she brought an ultrasound scan showing a right adnexal mass 6 cm X 4 cm with fluid in the Pouch of Douglas suggestive of ruptured ectopic pregnancy. She denied history of sexually transmitted diseases but volunteered that she had had more than 25 lifetime sexual partners and did not regularly use barrier contraception. The pregnancy was not planned and she was negative for Human Immunodeficiency Virus (HIV). She had no history of fertility treatment or previous abdominal-pelvic surgery. She smoked tobacco and drank alcohol.

**Figure 1:**
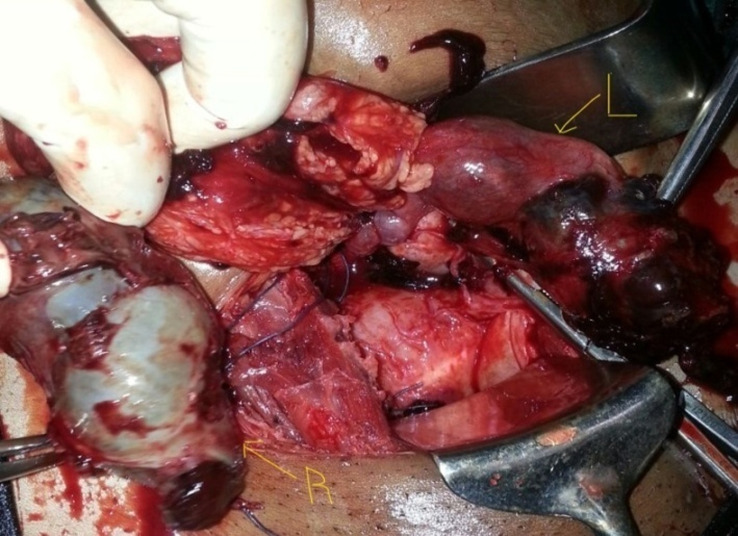
bilateral ruptured ampullary ectopic pregnancy at laparotomy, L-left, R-right tubal ectopic pregnancy

**Figure 2:**
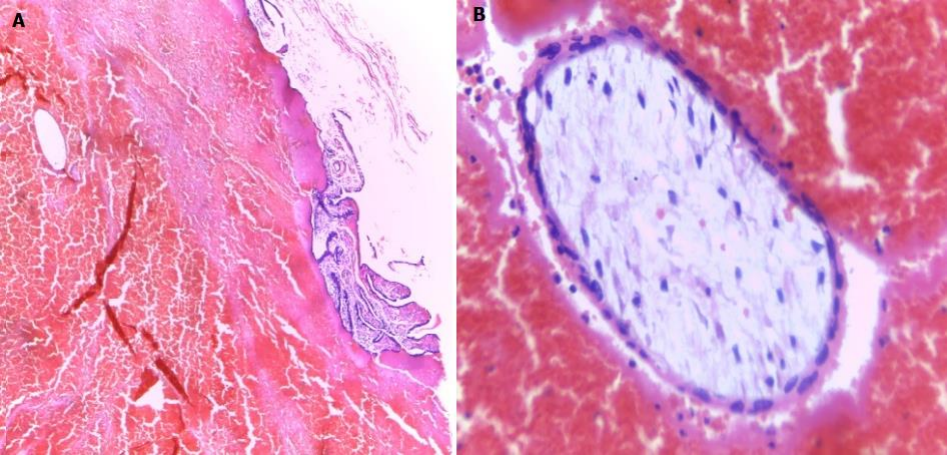
histology of the left fallopian tube; A) left fallopian tube-luminal blood clot with single chorionic villus at upper left corner, magnification X 40; B) chorionic villus in cross section showing immature (embryonic) mesenchyme core with surface trophoblast, background shows red blood cells, magnification X 400

**Figure 3:**
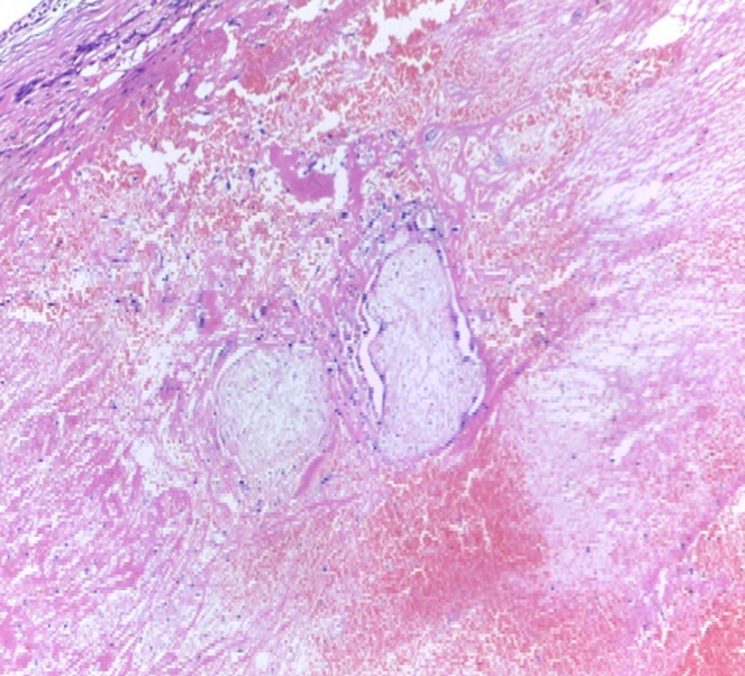
histology of the right fallopian tube, right fallopian tube wall (upper left) with luminal blood clot and two degenerative chorionic villi, magnification X 40

On examination, she was ill looking, very pale, BP 110/57 mmHg and pulse 110 bpm. The abdomen was mildly distended, tender, with guarding and rebound tenderness. Vaginal examination revealed minimal bleeding and cervical excitation tenderness with a right adnexal mass. A full blood count showed white cell count of 15x10^9^/L and haemoglobin of 6 g/dl. She was resuscitated with intravenous fluids, X-matched blood and after an informed consent was obtained, emergency laparotomy was done. At laparotomy there was 900 mls of haemoperitoneum suctioned and bilateral ruptured ampullary ectopic pregnancies (Figure 1). The uterus and ovaries were grossly normal, but none of the tubes were salvageable and bilateral salpingectomy was done. She was transfused three units packed cells, one unit intra-operatively after haemostasis was achieved and two units postoperatively. She was counselled on the operative findings, the benefits of using condoms consistently and cervical cancer screening. She recovered well and was discharged home after four days. She was reviewed two weeks later and at two months she had no complaints. Pathology results confirmed ectopic pregnancies with blood clots and placental villi in both fallopian tubes (Figure 2, Figure 3).

## Discussion

Spontaneous bilateral ectopic pregnancy is the rarest form of extra-uterine gestation and difficult to diagnose preoperatively [6]. Most patients with bilateral tubal ectopic pregnancies present with symptoms similar to those with a unilateral ectopic pregnancy and have similar risk factors. The most frequent findings are the triad of amenorrhea, vaginal bleeding and abdominal pain. Levels of serum beta component of the human chorionic gonadotropin (β-hCG) and the discriminatory zone are not reliable for patients with bilateral disease [4].

The case we report meets the criteria outlined by Norris: chorionic villi were demonstrated on histopathological examination of the tissue obtained from each tube. The majority of reported bilateral tubal ectopic pregnancy have one tube ruptured and the other intact, so conservation of at least one tube is undertaken [7,8]. However, in our case both tubes had ruptured, so bilateral salpingectomy was performed. Ultrasonography in our case failed to make the diagnosis and this is in agreement with other reports, which affirms that the use of ultrasound is not necessary to diagnose BTP [8]. Thus, the diagnosis of bilateral tubal pregnancy is usually made intra-operatively. In a case report in China, synchronous ectopic pregnancy was missed in the contralateral tube at laparoscopy [5]. The patient was re-operated five days later, after follow-up β-hCG continued to rise. Fortunately, the tube was un-ruptured and she survived after salpingostomy. This underscores the importance of identifying and closely examining both tubes at the time of surgery.

Several theories have been postulated to explain the occurrence of bilateral tubal pregnancies. Bilateral tubal gestation requires multiple ovulations to occur, the oocytes to be fertilized and implant at sites of tubal damage [4]. Another possible aetiology is transperitoneal migration of trophoblastic cells from one tube to another, which explains the finding of fetal tissue in one tube and only villi in the other tube [9]. Superfetation, is another possible aetiology, with fertilization and development of a second ovocyte in a woman who is already pregnant [9]. It is an extremely rare event, difficult to prove and is suspected when severe growth discordance is seen in a multiple pregnancy. Laparoscopic salpingostomy or salpingectomy is the gold standard treatment modality for bilateral tubal ectopic pregnancy although laparotomy may be indicated in unstable patients [3]. There are no case reports of BTP treated primarily by methotrexate.

Where tubal conservation has been done, careful attention should be directed to follow-up tests. Serial measurement of serum β-hCG is important to rule out the risk of persistent trophoblast until complete resolution [7]. Our patient, with haemodynamic instability, was not suitable for laparoscopic surgery nor salpingostomy as both tubes were extensively damaged. She was unique from the majority of the reported cases in that both her tubal ectopics were ruptured, as she had presented late, at 14 weeks. This emphasises the need for an early pregnancy scan which would have permitted diagnosis and intervention before tubal rupture and preservation of fertility. Rarely the diagnosis may be made on these preoperative scans as the case reported by Nitivi in India who also had bilateral tubal rupture [10]. The fact that our patient got pregnant unintentionally brings to the fore the need for all health practitioners to be vigilant in order to provide health education and offer contraception to all sexually active women and reduce the unmet need.

## Conclusion

Bilateral tubal pregnancy is likely to be missed pre-operatively even with an ultrasound scan leading to severe morbidity and potential fatality. Therefore, a careful examination of both fallopian tubes at surgery for ectopic pregnancy is critical to prevent secondary surgery, maternal morbidity and mortality.
